# Treatment of Traumatised Sexuality

**DOI:** 10.3389/fpsyg.2021.610619

**Published:** 2021-03-16

**Authors:** Elsa Almås, Esben Esther Pirelli Benestad

**Affiliations:** Department of Health and Sports, Institute for Psychosocial Health, University of Agder, Grimstad, Norway

**Keywords:** sexuality, sexual health, sexual dysfunctions, sexual behavior, psychological factors

## Abstract

Based on therapeutic meetings with individuals who have experienced sexual violence and abuse, the challenge is how do we help these couples to establish sexual relationships on their own terms, without interference of defence or coping strategies they have used to protect themselves against the overwhelming experiences of violence or abuse in the past? This article will focus on therapeutic work with such couples and how to interact with them and support their efforts to establish satisfying sexual relationships, based on sexological experience as well as experience from work with traumatisation. The basis for our treatment is a modified version of William Masters and Virginia Johnson’s approach. The technique of sensate focus is central, modified by trauma theory, including the understanding of dissociation, and the need to integrate memories from different levels: somatic, emotional, and cognitive. The traumatised client needs special attention to the experiences of predictability and safety and respect due to their history of being transgressed against. The therapists must be aware of the issue of dissociation; different dissociated inner parts can play different roles in the interaction between client and therapist. While couples therapy is a necessary frame for this therapy, the therapist often needs to work with issues unique to each individual. Each partner must be able to identify their own responses and their own sexual needs and preferences. It may therefore be valuable to have a co-therapist. The central goal is for the clients to identify responses to stimulation as a here and now experience in a setting that feels safe and welcome.

## Introduction

Traumatic reactions resulting from sexual violence and abuse have been a subject of therapeutic treatment for several decades, especially since post-traumatic stress disorder (PTSD) was introduced as a diagnosis in DSM-III in 1980 (APA, 1980). However, many therapies focus only on coping with the traumatising experience and conclude treatment once the original trauma has been processed, without helping the clients to establish sexuality on their own terms. Psychological treatment alone is only minimally effective in rehabilitation of clients’ sexuality, and it is therefore recommended that sex therapy be added (Maltz, 2002; Almås and Landmark, 2010; Landmark et al., 2012; O’Driscoll and Flanagan, 2016).

Additionally, too little attention has been paid to the silence imposed upon individuals who have experienced violence and abuse, which contributes to their sexual challenges. Silence may be imposed either by the abuser or by society (or both), given the general lack of openness regarding sexuality, and adding to *traumas of retention*. Feelings of guilt, and of having participated in the abuse, can contribute to retention of guilt and continued silence.

Treatment aimed to establish/re-establish adult sexuality on the individual’s own terms will be described in the following. The treatment approach will be based on modified sex therapy in combination with trauma therapy (Almås and Benestad, 2004a,b; Masters and Johnson, 1970; Kaplan, 1974; Hertoft, 1980, 2002; Langfeldt, 1993; van der Kolk et al., 1996; Schnarch, 1997/1998; Rothschild, 2000; Clement, 2002; White, 2004; Lundberg and Löfgren-Mårtenson, 2010; Porges, 2011; Ecker et al., 2012; van der Kolk, 2014/2015; Almås and Benestad, 2017). Some of these authors are Scandinavian contributors to the Nordic sexological tradition, which may be unknown to many, but which will be introduced below.

## Treatment of Sexual Problems

Sex therapy is based on physician William H. Masters and psychologist Virginia E. Johnson’s methods of treatment of sexual problems in couples (Masters and Johnson, 1966, 1970), and Helen Singer Kaplan’s modification of this theory towards the outpatient treatment of sexual problems among individuals as well as couples (Kaplan, 1974). In Scandinavia, these methods were introduced by Preben Hertoft and his colleagues at the Sexological Clinic at the National Hospital of Copenhagen (Hertoft, 1976, 1980, 1987), Thore Langfeldt in Norway (Langfeldt, 1993), and Per Olov Lundberg in Sweden (Lundberg and Löfgren-Mårtenson, 2010).

Preben Hertoft recommended that the treatment of sexual problems should be considered as an element of an interdisciplinary, holistic approach. Before implementing sex therapy techniques, in most cases, the client is provided a physical examination, and the relational and individual factors are explored. Sex therapy is based on a combination of knowledge about sexual physiology and sexual responses as well as methods for the modification of responses. In addition, many therapists find it necessary to consider dynamic issues related to the individual’s history of development, established patterns of sexual arousal, interplay between the partners involved, current stresses in the client’s environment, individual motivation and capacity, knowledge about, and attitudes towards sexuality in general, as well as the client’s ideas of the ultimate goals of the treatment (Hall, 2008; Brotto et al., 2012; Goodbout et al., 2020; Gewirtz-Meydan and Ofir-Lavee, 2021). The bio-psycho-social model described by Engel (1977, 1980) offers a useful perspective for many sexologists and has become the gold standard for sexologists.

## Preconditions for Sex Therapy

In order to establish a safe therapeutic space, the therapists must feel safe and comfortable concerning their therapeutic expertise as well as with the clients’ sexological challenges. Many therapists report that they lack a satisfactory level of professional competency regarding sexuality and, therefore, avoid discussing this aspect of their clients’ problems (Træen and Schaller, 2013). Many clients present symptoms of underlying, often hidden sexual problems and challenges, but avoid bringing these to the therapist’s attention, even as numerous clients wish for their therapists to broach the subject of sexuality (Almås, 1983; Træen and Schaller, 2013).

Clients may have different needs when it comes to talking about sexuality. We find it useful to introduce this possibility by providing careful permission to introduce individual needs according to the PLISSIT model (Annon and Robinson, 1976). In the model, Permission (P) is the first level to approach sexual problems. On this level, most sexual problems are solved simply by having permission to be sexual. The next level is Limited Information (LI), where more problems may be solved through conveyance of knowledge related to the challenges that the clients experience. Some clients will need Specific Suggestions (SS), which may include sexological counselling and basic sex therapy. Only a minority of all sexological clients will need Intensive Therapy (IT), where specialized psychotherapeutic or medical skills are required. Therapists must recognise, however, that all levels of intervention should be available, especially for traumatised clients.

Many clients are not ready for sex therapy, even if they seek treatment due to sexual problems, as observed, for example, in the following cases:

Sandra had been abused by her father, his friends, and her schoolmates during most of her childhood. She sought therapy because of lack of sexual desire. When we talked about the reasons for having sex, she said that she had never felt any positive sexual feeling in her body and that everything that had to do with sexuality was associated with degradation, pain, and nausea. “*For me, it is only violence!*” she said.

In such situations, extensive work is needed before it is possible to get close to the feelings of sexual desire or enjoyment. This should be pursued in individual therapy. It may be a way to go from violence to pleasure!

Anna had a painful condition in her pelvis since she was 13 years old. She had never had any positive feelings in her genital region; everything that she could sense was connected with pain. She sought therapy because she wanted to be normal.

When Greta was 7 years old, she was invited to touch her uncle’s penis in the bathroom. She was curious and touched it. Immediately she understood that she had crossed a boundary and that she could not run to her parents and tells them what had happened. Later, when she had the words for it, she said that was when she got the feeling that “*I was a whore!*”

John, at the age of eight, was taken aside by a “nice lady” who took him to the woods and abused him sexually. Later in life, John felt that he had taken part in something so abominable that, again and again, through emotion-driven trespassing actions towards others, he would prove to himself that he really was as mean as his intrapersonal narrative presented him to be.

Diana had experienced over and over again situations, where her mother, in many – at times subtle – ways, exposed herself to Diana and also touched her in ways that were violating. For Diana, this became so unbearable that as she grew up and got a clearer picture of her mother’s behaviour, she had to move her mother out of her sphere of significant others in order to shelter herself from the disturbing memories.

There are many narratives that tell various stories about sexuality. Some patients have been able to explore sexuality on their own terms, as feelings have emerged through accidental self-stimulation, exploration with other children or teenagers, falling in love, secretly exploring books and films with sexual content, or other sources of sex education, of varying accuracy. Alternatively, some, like Diana, find ways to protect themselves while also realising that they have a sexuality of their own that calls to be explored.

The therapist must be aware that the story the client has to tell may be surprising, unusual, involve shame and guilt, and even be a secret that the client has never dared to share with anyone. In order to build a therapeutic alliance, therapists must prove themselves worthy of being told this story. When one as a therapist gets invited to know such a story, one must regard it as a valuable gift to be treated with the utmost respect.

It is also useful to remember that when a therapist takes time to explore how the sexual history of the client has developed, they must realise that their therapeutic skills are important, that the therapist will require everything they know, and that they must fully value their therapeutic competence. It may be necessary to possess the *skills needed for intensive therapy* in accordance with the PLISSIT model. In addition, it is usually necessary to add *sexological knowledge* in order to work with bodily reactions, sexual responses, relational wounds, and self-hurt, and to help clients become aware of positive sexual feelings, acknowledge them, and, hopefully, be able to enjoy them.

## Avoid Retraumatisation

Individuals who started their sexual life by being traumatised will often fall into a pattern of recurrent traumatisation. It is important to identify how the trauma could repeat itself and ensure that the treatment does not turn into a repetition of the original trauma. There are examples, where individuals have experienced that the assigned treatment has staged revisitations of the trauma. In the worst of cases, this has been a reality, meaning that the therapist has actually been an abuser, but it is bad enough for the client to live with a subjective experience of not being able to escape the trauma, leaving them with the message, *You are an eternal victim!*

Revisitation of the trauma is more likely to occur in situations, where the rules of therapy are unclear, under circumstances that are secret, and in conditions, where areas of responsibility are not clearly defined. Traumatised clients are often extremely sensitive with regard to rules, limits, contracts, and mutual areas of responsibility. Many have been trained for years in alertness and are able to sense betrayal or neglect, even when these do not exist.

It is striking how some clients go from one trauma to another. The lack of an ability to shelter themselves from situations that may become threatening may be an important factor contributing to such repetitions. They tend to stay in situations when others would have left, being victims of an underlying need to satisfy other people’s needs. Sometimes they may experience others, including the therapist, as real or potential violators. Therapists, like anyone else, may become the victim of projected identification and take on the role of the violator; the likelihood of this increases when the therapists are not aware of their own countertransference. Retraumatisation may occur in the form of therapists’ lack of respect for appointments, abuse of power, unclear social lines between the therapist and the client, and in the worst case, sexual abuse. As far as the treatment goes, there are various challenges related to early sexual traumas. The body seems to live in denial as long as it needs to do so. Once sufficient safety has been established, and the ability to endure pain is sufficiently developed, the individual may recall traumatic experiences spontaneously or as a result of a random incident. Let us underline that we are sceptical about the therapeutic necessity of provocation of potential traumas with the help of hypnosis or guided association, as the therapist may well contribute to the development of false memories in such cases. Lack of autobiographical memory and/or of integrated cognitive schemas for the formation of opinions, continuous dissociation, identification as a victim, and experience of helplessness and betrayal may render the client highly susceptible to suggestions and constructed explanations that are out of proportion to the real incidents, which may have caused the problems. The therapist must wait until the client is ready to talk about the trauma. It is similar to a splinter in the finger: when the body is ready to get rid of it, it will come out smoothly and easily, accompanied by some infected matter.

In the initial acute phase of processing sexual abuse, there is usually no point in discussing the client’s current feelings toward sexuality. It is important to distinguish the story of abuse from that of personal sexuality. When individuals explore their former experiences of abuse, it is crucial for development in therapy that they place these experiences in the past and as belonging to the abuser’s lack of understanding of boundaries. In order for the client to explore their own sexuality, it is important that they feel the right to have their own boundaries and the possibility of both acknowledging and defining their own physical feelings and sexual needs. It may be difficult for many clients to identify the story of abuse as different from their own sexual story early in therapy.

Here, our focus is on sex therapy with couples, but many clients also require an individual approach. Sometimes it is necessary to stop therapeutic work with the couple in order to work with individual issues. Most often, the traumas took place long before the partner entered the abuse victim’s life. Hence, the partner must have “ice in the veins” and wait for the time to be ripe. Our approach focuses on developing new sexual patterns, different from the sexual experiences connected with abuse or violence, thereby avoiding the role of a victim. During treatment, the focus is shifted from the history of abuse to the story of sexuality on one’s own terms. We attempt to extract the individual’s own sexual story from their life story, and finally the story of the couple that is developing as an integral part of the relationship. Many clients who have been subjected to abuse and have received considerable treatment feel that attention has been focused on them as the source of all problems. As a result, they often feel responsible for any problem that the couple may be experiencing; they also carry the burden of the couple having to go to therapy. To avoid this, when we initiate sexual therapy, we emphasise that the treatment aims at processing the couple’s sexual interplay. When one partner has experienced sexual abuse, both partners may have been influenced by the original trauma in such a manner that inexpedient sexual patterns may have been established.

In order to begin sex therapy, it is usually an advantage if the client:

has identified and processed previous traumatic history,

is reasonably safe and free from high levels of stressors in the current life situation, and

is ready to establish healthy sexuality with their current partner.

Similarly, the therapist must:

feel comfortable talking about sexual issues,

have a satisfactory level of sexological knowledge and experience, and

be able to employ every aspect of their general therapeutic expertise.

It is also important to recognise the influence of the culture in which the client is situated. In therapy, family cultures may be important to explore, in order to identify unconscious patterns learnt by each partner in their respective families. For example, some clients are used to strong expressions of emotion, while their partners may experience these as signs of hostility and separation.

Further, culture often imposes silence on powerful emotions and experiences related to sexuality and that imposes an even greater silence on the experiences of sexual violence and abuse. Clients experiencing this process may therefore lack words, and it is important to be patient and allow the clients to find their own ways of expressing their experiences.

## Establish a Safe Space in a New Room

We regard the therapist’s office as a training arena, where it is safe to address difficult and challenging topics. Most people who enter the therapy room regard their life situation as critical and feel the need to establish a subjective territory, while at the same time experiencing their history of abuse as a dark force that has taken away their ability of self-determination. This dark force is often present in the therapist’s office and must always be clarified, defined, and defused or neutralised when it appears. For many, the therapist’s office is the first place, where they have felt safe to talk about themselves. Initially, the therapist guarantees safety, and the therapeutic work consists of assuring the client that this sense of security is developing and becoming a part of the client’s autonomy. The starting point for security is the therapist’s own level of expertise, professionalism, and respect for the client’s autonomy and integrity. An important part of this competence is to separate the individual’s story of abuse from the story of their sexuality.

Tom: *It’s good that this part of therapy takes place in a different room, it makes it easy to separate my stories – this is a completely different one*.

## Respect and Safety

It is taken for granted that respect and safety are to be the basis of all forms of treatment. Yet we have heard far too many accounts from clients who have dissociated in the therapy room and been unable to tell their stories. Instead, they have presented physical symptoms, aversive reactions, anxiety, depression, or aggression. Needless to say, this is often supported by the therapists’ ways of questioning, what the therapists overhear, and what they are able to extract from the conversation.

Jenny: *So many years went by before I could start talking about it. I can still feel the straps from the psychiatric ward around my wrists*.

Following a medicalised, psychiatric tradition, therapists have often been more concerned about symptoms and diagnoses than the personal stories behind the symptoms. Many clients tell stories about being medicated by either having a prescription, such as for Viagra, put in their hands with no further follow-up or having gotten various kinds of psychotropic drugs for curbing their reactions.

Tom: *I got a prescription for Viagra, and I thought at that moment,* “*Well, that’s the end of sexuality for this guy.*”

Safety means creating a climate in which it is possible for the client to leave behind a certain amount of the presently undesirable reactions that they have developed for self-protection. It also means creating a space for developing new stories, which affirms an individual’s ability to remain standing in spite of the storms they have been subjected to in the past. The development of these “stories of victory” occurs simultaneously with the clients gaining new perspectives on their own life.

## Clear Agreements and Boundaries, and a Clear Division of Responsibility

Individuals who have been subjected to serious abuse are often vulnerable and interpret unclear signals as signs of danger.

Jenny: *Since you have not brought up what we agreed to talk about last time, I’m starting to feel insecure. Maybe you do not know what you are doing?*

If the therapist hesitates to work out a plan for treatment regarding how the therapy will progress, the client might believe that their therapist lacks concern about their problems. If the plan remains unclear, the client may see this lack of clarity as an invasion of their privacy. In cases where the therapist does not assume clear responsibility for treatment and make it clear that communication on painful topics and sexual matters is welcome, the client might begin sparing the therapist discomfort by withholding unpleasant information – the same information that may be essential for creating a picture of the client’s real problems.

## Time

When is the right time to work with sexuality? For some clients, sexuality must be addressed early in therapy, while others need to establish a safe feeling of self and integrity in order to address sexual issues.

Emma: *I would never have been able to speak about this ten years ago, when I started therapy. It’s good to have gotten the chance to process it and create a life before I got to work on this.*

Kari’s partner: *It’s good that we did not get an appointment before now. Last year, she was so far down that she had a hard enough time just working through her depression.*

The development of the process at a safe pace takes time.

Tom: *It was only when I came here with my lack of sexual desire and you asked me about my sexual history that I realized I had to tell you about my abuse. It’s taken me more than forty years to get that far!*

Nora: *When I talked to the other participants in the group about sexuality, it was like opening up a secret room that we all wanted to enter but were afraid to go into!*

## Who Is the Expert?

Regarding processing sexual trauma and establishing new sexuality based on the clients’ terms, it is understood that the therapist is the expert. This expertise lies in the knowledge of treatment techniques, familiarity with the possibility of retraumatisation, and experience with the therapeutic development and maturation processes. Being an expert does not mean having knowledge about what is good sexuality for a particular client, nor does it mean having knowledge about how the particular individual will reach a goal that may be satisfactory to them. Rather, it concerns being able to relate to each individual’s unique story with knowledge regarding sexual development and the necessary therapeutic skills.

It is often necessary to use several therapeutic approaches simultaneously (Almås and Benestad, 1993). At times, we have observed new approaches developing through our interactions with the clients. For instance, during the treatment of an engineer, we tried to determine his approach to solving an apparently impossible problem by asking him what he did when he had a problem at his work? “I just add a new dimension,” he replied. This simple method became one we could use both with him and with many other clients.

## Entering Couples Therapy Through Individual Therapy

Individual therapy is used, where there are problems that concern the privacy of one of the individuals in the couple, for instance, earlier sexual experiences with other partners. Sometimes we have to stop couples therapy in order to focus on individual issues that may appear, like individual turn-on patterns, issues in the individual’s history, infidelity, or bodily problems. Usually we recommend that trauma therapy is concluded before we start working with sexuality in the couple; we also recommend a consolidation period between the two different treatment processes.

We often say that we do not treat the couple; we treat *individuals* who constitute couples. This means that our primary aim is not to keep individuals in partnerships, but to attempt for both individuals to leave the therapy room as intact individuals, irrespective of whether or not they continue as a couple. We find it useful to employ a systemic approach, where the system involves the body, the individual, the couple, and the culture to which they belong. We have used systemic models described by the biologist Ludwig von Bertalanffy (von Bertalanffy, 1976), as well as ideas developed by Humberto Maturana and Francisco Varela (Maturana and Varela, 1992).

We also find the bio-psycho-social approach described by Engel (1977, 1980) useful, as sexual problems might require expertise from different professions. This model acknowledges therapeutic resources and limitations, proposing the different kinds of experts who can contribute synergistically to each other’s expertise. For psychotherapists, it may occasionally be necessary to include information from gynaecological examinations, hormone analyses, general physical examinations, or psychomotor skills evaluations.

This was observed in the case of Eva, who expressed a strong sense of being unclean. “*Now, where are people cleaned?*,” the therapist asked. After a few exchanges, the answer was “*A priest cleans people*.” A wise priest was subsequently contacted, and the cleaning process commenced as the client also continued therapy.

Individuals who have learned about sexuality through abuse often have an extremely low level of consciousness regarding their own wishes and needs. In these cases, it may be necessary to work through a phase of individual therapy in order to raise their awareness of their wishes and needs.

We use what we call the S-O-I model for this type of work. The model relates to the interplay among three aspects: *subject*, *object*, and *instrument*. The model is set up as a triangle in which the three aspects create the three sides.

*The subject side* includes the individual’s own wishes, needs, ideas, driving forces, desires, and impulses. The *object side* describes how the individual presents oneself as an object to the world, how other people regard the individual as an object, and how the individual becomes an object to oneself. *The instrument side* concerns the individual’s instrumental functions: physical function, skills, and talents.

These three sides interact with each other. We often observe that the object and instrument aspects take up a great deal more space than the subject aspects. It is, therefore, often necessary to raise awareness of and expand an individual’s sense of being a subject. Among other things, this is accomplished by raising their awareness of their own sexuality: sexuality is to be *discovered*, sexuality is to be *accepted*, and sexuality is to be *valued* (Almås, 1987). The object aspects must serve the subject, and must, to a lesser degree, be controlled by real or imagined demands and forces coming from the outside.

The aims of individual therapy can be described as follows:

Subjective experience of sexuality.

Subjective experience and interpretation of physical sensations.

General consciousness-raising of personal sensory preferences.

Integration of sexuality into the individual’s personal history.

Discussion of guilt and shame.

Identification of the sexual culture: different perceptions of the meaning of sexuality including personal perceptions.

Identification of sexual stimuli: personal preferences.

Identification of oneself as a sexual subject: what kind of sexual person am I?

Experience of being the owner of one’s own sexuality.

## Sensory Work

We work on raising the client’s sensory awareness in both individual and couples therapy. Bodily senses are important preconditions for sexuality. Clients are given homework that includes getting to know their senses one by one.

Before we begin exploring the senses in sex therapy, we examine the client’s feelings about the sexual, sensual, and erotic culture around them. They are encouraged to ask themselves, “What does sexuality mean to me?” Some people find sexuality to be all about performance, that one must be a sexual object; many clients have no regard for themselves as a sexual subject with their own wishes and needs.

This form of “culture watching” is designed to raise the client’s awareness not only about their own but also about other people’s sexuality. At the very least, it gives them the opportunity to gain a broad spectrum of insight into the fact that sexuality and sensuality exist in them and in the world around them.

During the first sensory week, the theme is the sense of *seeing*: “what do I see, what do I notice, what do not I like to see, how do I make sense of my visual sensory impressions?” Many may surprise themselves, and sometimes they become surprised by their surroundings. This task gives us the opportunity to discuss sensory impressions, as individuals become aware of their preferences in a somewhat neutral manner.

What about *scent*? A scent could be far away or nearby. Exploring the sense of *smell* can include everything from perfumes, food, things, and other people to the smell of a partner’s clothes and body.

In the same vein, comparing food and sensual eating is more than a metaphor; it is a physical analogy. To explore the sense of *taste*, we ask, “What is your favourite dish?” If the answer is, “all food is equally good,” we know that we have a great deal of work in store regarding sensory consciousness-raising. People like to be on solid ground, where it is possible to use several senses including their own subjective experience. Food provides not only a safe opportunity to distinguish between good and bad but also to rank the good and the bad.

We continue by encouraging a week of being aware of *listening*: what do you like to listen to? What do different voices sound like? What music do you like? What can our ears comprehend of sexual culture and erotic expressions in the surroundings that are both close and farther away?

One of our clients had a special experience when it came to listening. She felt that her boundaries were trespassed when her neighbour made too much noise when she wanted to sleep before her night shift, and she asked him to be quieter. The surprising thing was that she was not even aware of boundaries when it came to other senses. If a man looked at her in a particular way, she made herself available for him. When it came to smell or taste, she was more aware of what other people liked than what she liked. She usually followed other people’s preferences, except when it came to sound. We found that the abuse had never been related to what she heard; there were no sounds.

The next sense to be explored is *movement*: what kind of movement do you like? In terms of workout, do you like running, swimming, or dancing? What kind of dancing do you like?

One of our clients found that she liked tango! She liked the music, the way the dancers dressed, and the way they moved. She started to dress more in a tango style, and, to her surprise, her partner followed her by developing a more relaxed style, using black t-shirts under his jackets, letting his hair grow a little longer, and taking tango lessons with her. This also became a route to their sexuality through the sensuality of tango!

The sense of *touch* is for many the most intimate sphere of sensation. In exploring touch, we ask our clients to explore what kinds of touch they like and dislike. What is comforting, what is offensive, what is loving, and what is erotic touching? Anyone who has been touched on other people’s terms needs time and awareness-raising in order to discover their own preferences regarding how, where, and when they like to be touched. Diana, whose mother had been constantly trespassing her boundaries, hated hugs, especially if the hugger was a woman.

Subjective sensory competency is the foundation of developing personal sexuality, and the senses themselves nourish an individual’s desire and the level of sexual arousal. The senses must be made conscious and trained in order to be used for the subject’s service, as sexual abuse far too often leads to the senses being used to satisfy other people’s needs.

## Couples Therapy

In couples therapy, where one partner has been sexually traumatised, we have found it useful to have two therapists. It might be necessary to provide a great deal of support and attention to one of the partners for limited periods of time during therapy, and it is therefore advantageous to have a second therapist who can focus on the other partner when this occurs.

It is important to emphasise that the two partners have their individual histories, which are the individual basis for interacting as a couple.

It is a general experience that there must be a basic attraction between the partners for sex therapy to be meaningful. Many people want to live together as couples without having the necessary emotional preconditions. Therapy offers to create conditions conducive for individual wishes and needs to be met. However, if the basic wish to be intimate is not present, no amount of therapy can help to develop a satisfying sexual relationship.

## Sexual History, Different From Abuse History

Couples therapy begins with a discussion with both partners in which the therapists explain the therapeutic procedure and discuss the preferred therapeutic options with the couple.

At this stage, both partners are encouraged to write down their sexual histories and send them as letters to the therapists. We make it clear that they are to write their own sexual story, and that these stories are personal, private, and not to be shared with each other. In other words, they are not supposed to read each other’s letters. Even in good couple relationships, it is important to be allowed privacy, especially when it comes to one’s sexual history. Clients are asked to send their letters to the therapists to allow sufficient time to read them before the subsequent session. We also tell them that these letters are their property and that they can ask to have them returned when therapy has concluded.

For those who have been subjected to sexual abuse, we make it clear that they are to write *the history of their sexuality* as *separate and different from their history of abuse*. During the development of their personal history, they can also use aids, such as pictures and other stories, from their family or friends. We usually need to spend some time asking them what kinds of sexual bodily feelings they experienced before the abuse began, their common physical sexual responses, and the times they had fallen in love and enjoyed their own sensory experiences and preferences.

One of the great taboos that therapists may have to deal with is that clients may have been exposed to *forced learning*, that is, that they have responded sexually in the abuse situation and in that way learned a sexual arousal pattern that may include fantasies of violence and/or abuse. When they feel safe, some clients disclose sexual arousal patterns that they regard as loathsome, associated with both guilt and shame. Secret fantasies can be powerful but can lose their power when they are shared. We regard arousal patterns as a language; we learn from what we are exposed to. Sometimes we need to learn a new language, for example, by developing new fantasies. One of the main goals of sex therapy is to leave unwanted patterns behind and develop new ones.

Some of our clients never experienced sexuality on their own before the abuse began. In these instances, we can use the exploration of preference through their senses and also develop and explore new sensory experiences in order for them to establish their own sexuality.

For example, Sandra felt that having sexual feelings in her body was a bodily betrayal because she felt that these feelings were within the domain of the abuser. Slowly she learned to have experiences through her senses, including sexual feelings that she could recognise as her own.

The next step in couples therapy always consists of individual sessions with one therapist and one client during which we discuss the individual’s sexual life story. It is important that the therapist is careful not to slip into the history of abuse and instead helps the client to separate the two stories.

During this conversation, themes may be clarified, and it is also made clear what shall not be shared during the sessions where the couple is together, even if both therapists need to be informed. One obvious problem arises in the form of instances where one or both partners have a secret sexual partner on the side. In such cases, this must be discussed, and other relationships ended before continuing therapy.

## Modification of Sensate Focus

When we are ready to move forward, we meet with the couple again, and provide instructions regarding sensate focus. Sensate focus is a therapeutic exercise developed and described by William Masters and Virginia Johnson (Masters and Johnson, 1970). When working with couples that have been traumatised, this exercise usually needs to be modified (Courtois, 1997).

The risk that traumatised individuals will slide back in time (*time slide*) when touched by their partner and make associations with abuse is often quite high. We, therefore, recommend that the here-and-now orientation be strengthened by practicing sensate focus with open eyes. It may also be necessary to take short breaks in order to orient oneself to the here-and-now situation. The partners learn how to reaffirm their presence, distinguished as a preferred partner. In order to strengthen the contact between the physical and the mental processes, we also recommend that they take turns writing letters and words on each other’s backs, in order to strengthen the relationship between cognition and body.

Time confluence, or *time sliding* may be clarified by asking, for example, “*How old do you feel when these feelings of fear or anger come over you?*” Not only do we make distinctions in time, but we also attempt to distinguish between “*the adult I*” and “*I as a child*”: “*If you slide backwards now, do you have an adult present with you – is it the adult I that is present?*”

## Establishing Sexuality on One’s Own Terms

Working with adult sexuality involves relating to the physical memory patterns that are present. Preben Hertoft describes sensate focus as *a probe to catathymic areas*, meaning that individuals easily come in contact with their emotionally vulnerable areas through these exercises.

When working with sexually traumatised individuals, it is important to place extra emphasis on the here-and-now experience, because every sexual situation can easily send the individual who has experienced abuse back to the traumatising experience. Even a light physical touch can evoke painful past images or conflicted physical sensations, as can smells, sounds, and other sensations. Many individuals describe feeling uncomfortable having another person’s body touching their own. The sight of a penis can evoke painful memories, as can the smell of semen; even a partner’s naked body can awake painful associations. Learning to focus on what one is actually feeling here-and-now during sensate focus is, therefore, a significant part of therapeutic work.

## Working with Language

The client’s language is often fumbling in nature:

*It’s difficult to find the right words – difficult to express them. It’s been difficult for a long time – have not understood it. Touch and intimacy in a purely physical sense is difficult. I start physically rejecting the other person*.

The therapist’s task is to help the clients to develop their language using complete sentences. The therapist’s office becomes a training ground for the development of sexual language; as a result, therapists must be able to move among several linguistic modes. We use concrete words for sexual organs and sexual functions. At times we use refined language, at other times earthier expressions. For example, we might say, “*there is a huge gap between no sex at all and intercourse*,” a sentence, which in another context or in relation to another individual may be phrased as, “*There is much between nothing and fucking*.”

## Use of Metaphors

Not least because of guilt, shame, and the fact that many individuals who have been subjected to abuse remain stuck in a victim’s role, allusions play a significant part in the language we use. By using a language where we say, “*the abuse has nailed you to a cross, and eventually your partner also came to hang on these nails*,” thoughts are awakened not only about the victim but also about where the guilt should be placed. Language and metaphors are offered to clients. Significantly, metaphors are meant to clarify or open, and not to circumvent.

*Circumventing metaphors*: Such as those used in films from the 1950s, where waves breaking on the sands were used to illustrate sexuality.

*Clarifying metaphors*: Using tastes typically appreciated more by adults than children, such as coffee, alcohol, or olives, as metaphors, we emphasize not only that sexuality is to be learned but also that physical maturation is necessary for this to occur.

*Opening metaphors*: These serve the cause of reflection. We consider it essential that clients receive recognition regarding how we comprehend them and their problems. Opening metaphors are a type of feedback in metaphorical format about what we have understood with respect to what clients have presented to us. For example, we might say, “*What I’m hearing makes me think about what it’s like to bake bread: The dough has to rise before it can be put in the oven*.” When a client either grasps the metaphor or stresses that it is not appropriate to the situation because actually, the situation is such and such, the metaphor has served an opening, consciousness-raising purpose. For some clients, metaphors make no sense, and we just avoid using them.

## Distinguish Between Here-and-Now and Back-When

When turning one’s attention to the individual’s capacity for mastery in relation to their current life challenges, it is wise for the therapist to focus on the here-and-now in working with the problems, as the client’s ability to approach more sensitive parts of their personal history improves. If the therapist moves forward too quickly, the client might withdraw through depersonalization or dissociation, their level of anxiety may increase, and they may go along without truly being present. Ideally speaking, one should move forward so quickly that the individual affected is barely aware of having limits unpleasantly exceeded and without being overwhelmed by the pain this implies. It is essential that

The therapist establishes a safe feeling of a here-and-now situation.

The client be safe in the knowledge that the trauma will not repeat itself.

The client feels like an adult who is different from who they were as a child.

The client feels this difference verbally, emotionally, and physically and is always be aware of the possibility of time sliding.

## Interaction With Partner

It is important to not only acknowledge that abuse also affects one’s partner, but also raise awareness regarding precisely *how* this happens (Hendricks and Hendricks, 1992).

It’s so difficult getting through to her. It’s like she sees me through a stack of slides that represent her abusers – her father, other men. It’s like she does not see me, who I am.

The individual who has been subjected to extensive and early sexual abuse often notices that the part of their sexuality that comprises deeper feelings becomes filtered through their experience of abuse.

Some clients may have had positive sexual experiences before becoming established with a regular partner and feel that their problems first came to the surface when their relationship moved into a deeper phase. The partner may, therefore, start feeling responsible for their problems:

But my partner has not reacted that way to previous partners.

It is a normal part of our experience with the outside world that what we undergo in the present is affected by our previous experiences. A pleasurable experience may be intensified by previously pleasurable experiences; a feeling of sorrow may be intensified by previous sorrows. Sensate focus is a potent tool for distinguishing feelings that are connected with former, negative experiences from the purer experience of what is happening *now*. Initially, a partner often gets regarded as the abuser or a person who, in some ways, resembles the abuser. During the course of therapy, however, the partner becomes extracted as a unique individual who is different from the abuser.

It’s like his body is becoming more like my own, I can see myself in it and love it.

It’s two different things – what happened back then has nothing to do with this.

It’s important we see that sexuality is much more comprehensive than just intercourse; we notice that it comprises a much larger share of our emotional life.

Sometimes we have to schedule individual sessions in order to process portions of the abuse that led to traumatisation. When this occurs and couples therapy is postponed, the partner may feel set aside, feeling that once again, everything in their relationship concerns their partner who has been subjected to abuse. At this point, it is important to make them feel secure that this is a brief interlude during which the traumatised partner has to do more work in relation to their own past before the couple’s story can be further developed in the therapist’s office.

## Working With Guilt and Shame

Strong emotions of guilt and shame are often associated with repressed feelings. For example:

*There’s something wrong with me that I do not dare talk about*, or

It’s my fault that it happened; if the others had known this, they would’ve blamed me, too.

It is important to expose these emotions and talk about them, but not necessarily explore them in depth in this setting. Many clients have never dared to talk about these feelings with others. Nonetheless, whenever shame is brought to awareness, clients often see how unreasonable it is. It is easier to see that the abuse did not happen because there was “something wrong” with someone, but because the abuser had the power to take advantage of the situation.

An abuse victim may feel shame and guilt because their body sexually responded to stimulation during abuse. The general idea is that because the body had a sexual response, this must have been willed by the victim. The client must learn that the body can react without subjective participation; it can even react with orgasm during rape. This split between physical responses and subjective experience has been described by Laan et al. (1995) and discussed through a systematic review of research (Chivers et al., 2010).

Feeling overpowered and powerless can be especially hurtful when the individual who has been subjected to abuse is a man. Society dictates that men are not supposed to give in to power, even when they are truly being overpowered. It is often necessary to go deeper into our cultural understanding of what it is to be a man in order to provide the client space and permission to be powerless in a situation where he had no other option, and additionally, where he perhaps had a great need for adult contact.

Guilt often involves self-accusations: *Why did not I say no? Why did not I leave?* The clients project their adult capacity onto the child. We and they must recognise that they lacked the ability to get away at that time. During such cases as this, empty chair exercises may be useful. These exercises derive from gestalt therapy; in them, one distinguishes between the adult “I” and the childhood “I” in order to create communication between the two.

It may also be valuable to see the unique area of vulnerability that may be present before and while the abuse was taking place (Wyatt et al., 1993). It is common that feelings of guilt and shame disappear once the client understands the abuser’s ability to pick them out precisely as a person with an especially great need for contact and safeguarding at that particular place and time.

We have chosen a therapeutic strategy of maintaining a certain distance with respect to feelings of guilt and shame. We regard them as feelings associated with traumatisation, not with the individual who has been traumatised. By identifying these feelings in the here-and-now situation, we risk contributing to time-confluence and thereby mix up the abusive history with the current sexual experiences.

It is quite often obvious that the abuse is felt to be a kind of powerful “infection,” an unmanageable dark shadow or heavy burden in the lives of those affected. A victimized person may be treated like “a fragile egg,” which others must be careful not to handle too roughly. We can, through these examples, see that the abuse is infectious, and an increasing number of people become *nailed to the same cross*. As time passes, more and more people are hanging on these nails. In therapy, we see that traumas can be handed down through generations: no one remembers any longer when the trauma started, but feelings of shame may be transferred from one generation to the next.

## Development in Therapy

The therapeutic process is often slow, but can, at times move very quickly. There must be time for maturation, building alliances, acknowledgement, reflection, and epiphanies. In order for new learning and acknowledgement to take place, new mental and physical processes must be developed and integrated, new synapses developed in the central nervous system, and new patterns of reaction and action formed (Rossi, 1986; LeDoux, 1996; Aleksander, 1997; Damasio, 1999, 2010/2012).

Sensate focus is gradually expanded in conjunction with both parties’ experience of being safe. In most cases, we follow the classic pattern in the instruction of sensate focus: first, caressing the entire body with the exception of breasts and genital organs, thereafter including breasts, and later on, outer genital organs, then undemanding intercourse (the penis is in the vagina, without moving – in heterosexual couples) before they reassume control of their own sexuality (sometimes this even happens without the therapists having given instructions to move forward).

In some instances, we have deviated from this pattern or made variations on themes. However, we have experienced that too great a deviation can result in our giving in to clients’ resistance and that we as therapists lose control. The desired effect remains elusive because we become trapped by the clients’ inexpedient power struggles.

Examples of deviations that have worked in a positive manner:

*Caressing certain body parts:* This has been justified by a situation in which it was impossible to find enough time for full sensuality training. In order to have any possibility of physical contact, this became a technique that the couple developed themselves and eroticised to a high degree.

*Classified boundaries:* The couple was given charts of the human body and the woman, who in this case had been subjected to abuse, created her own boundaries regarding where she felt it was safe to be touched by her husband.

Examples of deviations, which, although they did not work at an optimal level, were perhaps necessary:

In a case, where it was the husband in a heterosexual couple who was traumatised, *the couple agreed that he will always be the one who takes initiative to do the exercises:* This was justified by the fact that she always had to take initiative earlier, and that she had no motivation to give him something positive without knowing that she would receive something in return. The problem with this solution is that we, as therapists, may become part of the couple’s power struggle, and might lose therapeutic freedom with respect to creating lasting change.

While it is important for therapists to show respect for the abuse itself, it is equally important that they do not dwell on the consequences of it when there is no longer any threat of abuse. This is a significant part of the therapeutic process. In order to move forward, it is necessary to make it clear that there is no danger of the abuse being repeated. It is our task as therapists to make sure that we do not become responsible for new episodes of abuse. It is equally important that processing the abuse itself during previous rounds of therapy leads to a safe life situation for the client. While one is leaving the victim’s role behind, the alternative positive story, the story of victory must be emphasised:

It’s like sinking down and looking at a new, beautiful underground world where both she and I are present.

It’s incredibly wonderful to see her pleasure.

At the close of therapy, we would like the following goals to have been achieved:

Acceptance of sexual feelings as they arise here-and-now.

Feeling of owning one’s own sexuality.

Well-functioning boundaries during sexual interplay.

Positive feelings about sexuality.

## Conclusion

Treatment aimed at re-establishing sexuality is ordinarily implemented when the traumatic experience has been processed and a safe life situation has been established and consolidated. Sometimes our clients bring up the need to work with their sexuality; sometimes they are happy that we ask them. Some clients come for sex therapy after finishing trauma therapy with other therapists, because they want to experience satisfying sexuality.

During couples therapy, both partners, as individuals, are at the centre of therapeutic interventions. While most principles from sex therapy may be used, it is necessary to also draw on principles from trauma therapy and body therapy. This will involve working with the interaction between cognitive, emotional, and bodily reactions. Trauma theory is of help in entangling complicated responses that may once have been a way to survive, but eventually have become obstacles to good sexual experiences. Therapists must pay extra attention to the danger of retraumatisation. Many clients who have undergone retraumatisation from healthcare providers show an admirable amount of trust by entering into a new round of therapy.

It may be useful to modify the instructions of sensate focus to avoid time sliding and contribute to re-learning sexual emotions and reactions.

It is fundamental to distinguish between back-then and here-and-now, maintaining the difference between these two perspectives at all times. Clients’ experiences of acknowledgement are often expressed as statements like “*Oh my goodness, it happened twenty years ago!*”

It is important as well that clients themselves use appropriate words to describe old experiences and new response systems, see how they differ, and assume ownership of the new responses developed through therapy. The therapist’s task is to actively make the conditions optimal for these new experiences and to curb reactions that belong to their clients’ former abusive situations. Finally, it is important that therapists create opportunities for change in the therapy room. There is a type of magic that we, as therapists, simply must respect: *If an Angel passes through this room, it must feel welcome!*

## Data Availability Statement

The original contributions presented in the study are included in the article/supplementary material, further inquiries can be directed to the corresponding author.

## Author Contributions

EA and EB: development of the therapeutic approach, discussion on principles and learning from therapeutic practice, implementation of theory and research. EA: lead author. EB: second author. Both authors contributed to the article and approved the submitted version.

### Conflict of Interest

The authors declare that the research was conducted in the absence of any commercial or financial relationships that could be construed as a potential conflict of interest.
